# A Knowledge-Navigation System for Dimensional Metrology

**DOI:** 10.6028/jres.107.017

**Published:** 2002-04-01

**Authors:** Howard T. Moncarz

**Affiliations:** National Institute of Standards and Technology, Gaithersburg, MD 20899-8200

**Keywords:** dimensioning and tolerancing, dimensional metrology, knowledge navigation, manufacturing training, VRML

## Abstract

Geometric dimensioning and tolerancing (GD&T) is a method to specify the dimensions and form of a part so that it will meet its design intent. GD&T is difficult to master for two main reasons. First, it is based on complex 3D geometric entities and relationships. Second, the geometry is associated with a large, diverse knowledge base of dimensional metrology with many interconnections. This paper describes an approach to create a dimensional metrology knowledge base that is organized around a set of key concepts and to represent those concepts as virtual objects that can be navigated with interactive, computer visualization techniques to access the associated knowledge. The approach can enable several applications. First is the application to convey the definition and meaning of GD&T over a broad range of tolerance types. Second is the application to provide a visualization of dimensional metrology knowledge within a control hierarchy of the inspection process. Third is the application to show the coverage of interoperability standards to enable industry to make decisions on standards development and harmonization efforts. A prototype system has been implemented to demonstrate the principles involved in the approach.

## 1. Problem Statement

Dimensional metrology is the science of measurement based on length. To fully understand the subject, a broad knowledge base that includes the measurement process, the language of measurement, devices, standards, traceability, and statistics is necessary [1]. Dimensional metrology is important because it is the basis for making parts correctly. Unfortunately, confusion in the correct application of dimensional metrology is common [2].

Among components of the knowledge base, two parts include (1) geometric dimensioning and tolerancing (GD&T) and (2) the overall inspection process. These represent two different perspectives; GD&T is the basis for some of the specific processes within the overall inspection process.

### 1.1 Geometric Dimensioning and Tolerancing

GD&T is a method to specify the dimensions and tolerances of a part so that it will meet its design intent, often to mate with other parts. Tolerances need to be specified tightly enough so that the part will “work” (i.e., meet the design intent); they need to be specified loosely enough so that the part can be manufactured at a reasonable cost.

The information required for GD&T and a symbology to communicate it on a part drawing have been standardized by the American Society of Mechanical Engineers (ASME) in ASME Y14.5M-1994 [3] (and referred to in this paper as Y14.5 for short). A similar system for GD&T has been developed by the International Organization of Standardization (ISO) as a set of standards [4]. However, we will focus on the use of Y14.5 here.

A large store of information is contained in the Y14.5 standard to guide the user on how to specify different types of tolerances and how to use the proper symbology. The subject is difficult to master because it is based on 3D geometric features and relationships that are difficult to visualize from textual descriptions, even when supplemented with 2D static figures. Also, when trying to interpret a particular tolerance and symbology, supplementary information is often useful but is not readily available without further page flipping and searching through the standard and other references. To fully convey the definition of the standard is difficult; to convey a deeper, intuitive understanding of it is much more difficult. However, that is the level of understanding necessary for a practitioner of GD&T.

### 1.2 Interoperability Standards

Analyzing the accuracy of a part based on tolerances is only a portion of the inspection process. That process includes inspection planning, data preparation, inspection execution, data acquisition, results analysis, and, finally, either acceptance of the part or feedback of the results to adjust an errant manufacturing process. These processes are supported by many software applications, including those that are incorporated into machine tools, e.g., numerical code execution systems. The entire system is most effective if the software applications are seamlessly integrated together at the information interfaces. Interoperability standards defined at the interfaces provide that capability.

Interoperability standards enable a manufacturing company to create a “best-of-breed” system, comprised of applications individually selected to best meet its needs and that can be integrated together within the system. The standards specify information exchanges among the applications to meet particular requirements. The challenge for standards’ developers is to specify a minimum set of standards to provide coverage for the information exchanges required that will also enable integration for the full range of software applications presently available and likely to be available in the future.

**Fig. 1:**
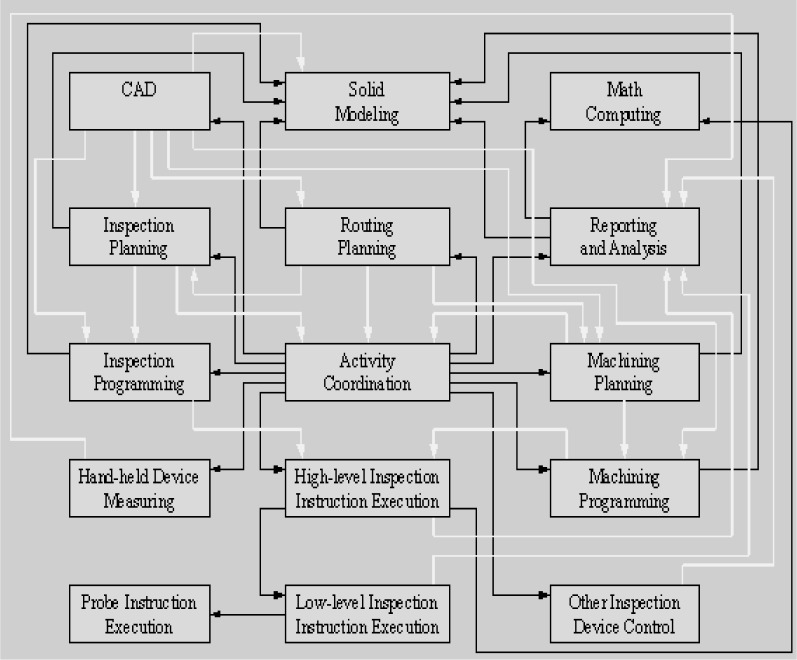
Modules and interfaces in a dimensional metrology system. Active interfaces shown in black, data interfaces in white.

A compilation was made of all of the possible interfaces in the dimensional-inspection process, and an assessment was made of the standards in place or under development to satisfy those interfaces [5]. Figure 1, from Ref. [5], shows the processes and information exchanges that were identified. (Note that active interfaces are defined as command-status interfaces in the reference.) The assessment indicated a large tangle of standards that included redundancies and conflicts where the domains of multiple standards overlapped and gaps where there was no coverage at all.

A large store of information is contained in and associated with the compilation and assessment in Ref. [5]. It is important to have a clear understanding of that information and its nuances, e.g., why certain information items are specified in certain standards but not in others that seemingly overlap the same processes. The assessment is large and complex and difficult to present clearly, particularly in the static format of a report. However, a clear communication of the assessment would help industry to prioritize its resources (along with government and academia collaboration) to develop and harmonize the standards required. If that could be accomplished, the market for applications supporting the inspection process could grow more efficiently.

### 1.3 Problem Summary

Dimensional metrology is an important subject but difficult to master for two main reasons. First, it is based on complex 3D geometric entities and relationships. Second, the geometry is associated with a large, diverse knowledge base that has many interconnections. Understanding the knowledge and the interconnections is necessary to master the subject.

This paper presents an approach to address the problem and describes a prototype system that was created to demonstrate the approach.

## 2. Solution

The goal is to provide an intuitive feel for different types of tolerances and to allow an intuitive access to a diverse knowledge base of dimensional metrology information. This goal leads to a novel approach that combines several aspects. The main idea is to create a knowledge domain that is organized around a set of key concepts and to represent those concepts as virtual objects that can be navigated with interactive computer visualization techniques.

The approach can be applied to the dimensional metrology domain to enable several applications. First is the application to convey the definition and meaning of GD&T over a broad and comprehensive range of that domain as represented by the Y14.5 standard. Second is the application to provide a visualization of a control hierarchy of the inspection process with links to dimensional metrology knowledge. Third is the application to show clearly the coverage of interoperability standards within the inspection process to enable industry to make intelligent decisions on standards development and harmonization efforts.

A primary challenge is to choose the key concepts wisely, including their representation as virtual objects. They should be as independent from each other as possible and should enable a wide and comprehensive coverage of the subject domain. For the concept to be useful in this approach, its virtual representation needs to comprise a decomposition into sub-concepts that distinguishes among information items in the knowledge base at a sufficient resolution to satisfy the applications needed.

The key concepts were chosen to address the three applications described above. The concepts are “part,” “tolerance entities,” “inspection process,” “interfaces,” “inspection device,” and “machining errors.” These are described below.

### 2.1 Part

The part is the final form of the initial workpiece. It has been specified to meet the design intent of the designer for function, manufacturability, etc. The part can be decomposed into features to serve different uses including functionality, manufacturing, inspection, etc. The features can be dimensioned and toleranced, and some features serve as datums.

A collection of parts can be carefully selected such that the features represent both an ample collection of manufacturing processes that were used to make them and include many of the dimension types and tolerances specified in Y14.5. Alternatively, one or more test parts could be defined specifically for this collection. Hence, the part is a main concept that can be associated with a great deal of manufacturing knowledge related to GD&T.

**Fig. 2:**
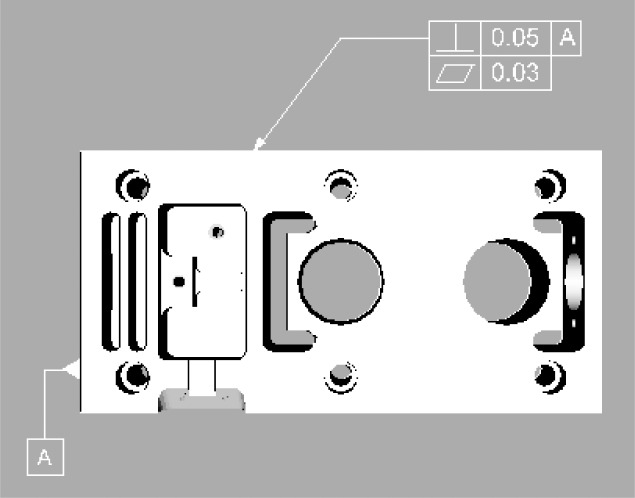
3D model of a test part oriented to show top view. GD&T callouts are also shown.

The part is represented in our knowledge system as a 3D virtual object of its shape. In addition, 2D dimensions and tolerances in Y14.5 symbology can be included as part of the 3D part, and displayed when the virtual object is rotated so that the 2D view of the part they are associated with is facing the user (Fig. 2).

### 2.2 Tolerance Entities

**Fig. 3:**
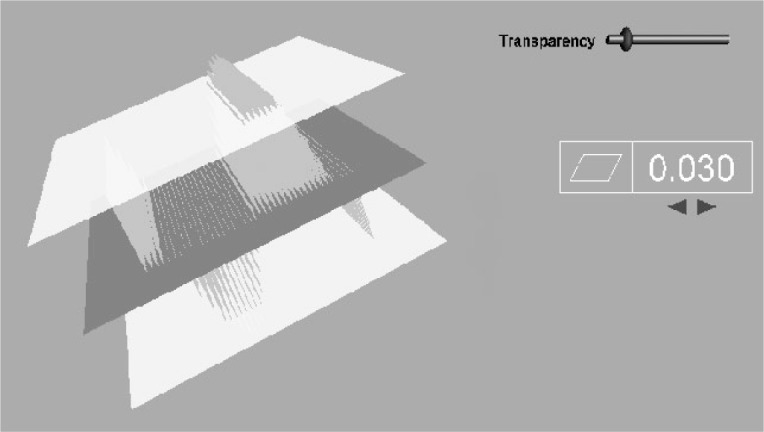
Flatness tolerance.

When a tolerance is selected from the part object, the entities associated with that tolerance are displayed. They include the feature toleranced, the datum reference frame or frames (if necessary), the tolerance zone, and the GD&T symbology of dimensions and feature control frame. The tolerance zone represents a boundary that separates in-tolerance and out-of-tolerance conditions. For example, for a flatness tolerance (Fig. 3), the tolerance zone would be the volume between two flat surfaces in 3D space that were spaced on either side of the nominal location of the toleranced surface by a distance of the tolerance value specified. The feature control frame succinctly specifies the information that describes the tolerance of the feature. It includes the type of tolerance (e.g., flatness, position, etc.), the tolerance value, the datums that may apply and the material conditions applied. (For example., maximum material condition, or MMC, is the “condition in which a feature of size contains the maximum amount of material within the stated limits of size, such as minimum hole diameter, maximum shaft diameter” [3].)

For this project, the feature may be represented as a set of one or more planes and/or cylinders, depending on the feature modeled (whether a hole, peg, slot, part side, etc.). Actual (or simulated) measurement points can be plotted in the feature space.

The set of feature, tolerance zone, and plotted points can be manipulated together to view the relationships of these entities from any viewpoint. The transparency of the tolerance zone can be adjusted from invisible to opaque to better view deviations of the feature that cross through the tolerance zone. The feature can be moved and oriented by the user to see whether the whole feature can fit within the tolerance zone indicating an intolerance condition. Also, the tolerance value in the feature control frame can be adjusted (by the arrow heads below the value 0.030) which causes the tolerance planes to move together or apart as the value is decreased or increased. In Fig. 3, simulated points are plotted as straight line deviations (“whiskers”) from the nominal feature plane in the center to represent a sine-wave surface. The surface is seen passing through the tolerance zone in the top right indicating the feature is out of tolerance.

By direct interaction with the tolerance entities, the user can obtain an intuitive feel for the meaning of different types of tolerances and how those tolerances are determined. The intuitive feel can be supplemented by displaying appropriate text and diagrams in other windows^1^ for the particular tolerance being studied.

### 2.3 Inspection Process

The control of the inspection process can be represented as a hierarchical task decomposition. That means that a high-level command is decomposed into simpler commands at each successive level of the hierarchy. For example, Messina, et al. [6] developed a demonstration implementation of an open-architecture, knowledge-based controller for an inspection workstation (IWS). In their IWS, the tasks for the control of the inspection process are decomposed, from the top, as manufacturing cell, workstation, equipment task, elemental move, primitive move, and servomechanism. A command from the cell to the workstation level to inspect a particular part is decomposed, ultimately, to commands to the servo controls of a coordinate measuring machine. In addition to commands and statuses sent up and down the control hierarchy, data are retrieved and stored at each level, measurements are taken at the lower levels and processed up the hierarchy, and judgements are made to intelligently account and adapt to the measurements taken after comparison with the world model (i.e., the currently estimated state of the system) while the inspection is in process. Much of the diverse information involved in the inspection process can be associated with a representation of the task decomposition of the IWS control process [6].

A 2D diagram can represent the task decomposition and even show the commands, statuses, and data flows, although the density of that information would be great. If you add in the interface standards and attempt to show how they are associated with the already dense information, it is evident that two dimensions are not sufficient to show that information clearly. Consequently, it was decided that three dimensions can show more information and show it more clearly. The thought process described in this paragraph led to the idea of “concept planes.”

“Concept planes” is the idea of arranging 2D diagrams that are hierarchically structured and related to each other in a stack so that the relationship of a component in one plane relative to a component in another can be inferred by their spatial relationships. The whole stack can be rotated and various components and planes can be made invisible to allow the individual components and spatial relationships among them to be seen. Any component may be selected to retrieve the information linked to it and display it in another window.

**Fig. 4:**
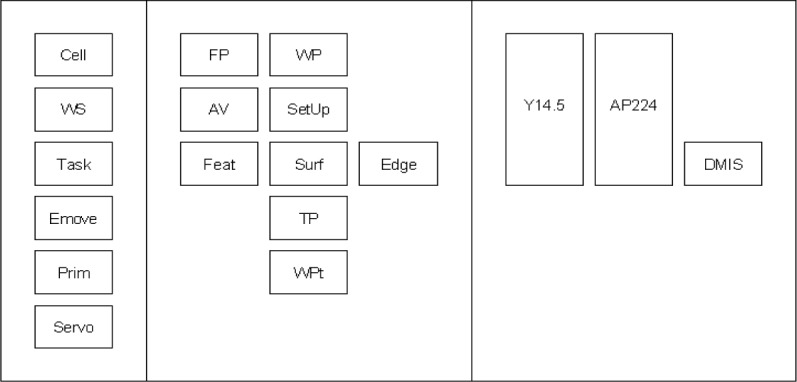
Individual concept planes for the inspection process.

Three concept planes are used to implement the concept described here for the inspection process. The three individual planes are shown in Fig. 4. In the left frame is the IWS control hierarchy, consisting of Cell, WS, Task, Emove, Prim, and Servo, which are acronyms that correspond to the control levels specified above. The figure could have included the commands sent down the hierarchy and the statuses sent up in response but were excluded here for simplicity. The middle frame shows the information stored or retrieved at each level of the control hierarchy. The information includes the final part specifications (FP), the workpiece (WP), access volumes (AV), setup data (SetUp), features (Feat), surfaces (Surf), edges (Edge), target points (TP), and intermediate points along the path that are referred to as way points (WPt). The right frame shows three of the interoperability standards used in the inspection process. The standards include the dimensioning and tolerancing standard, “Y14.5” [3], the standard that includes representation of machining features, “AP224” [10], and the dimensional inspection standard, “DMIS” [7]. Note that the Y14.5 and AP224 standards are elongated to fit in more than one control level to indicate that they are used to support information exchanges in multiple levels of the control hierarchy.

**Fig. 5:**
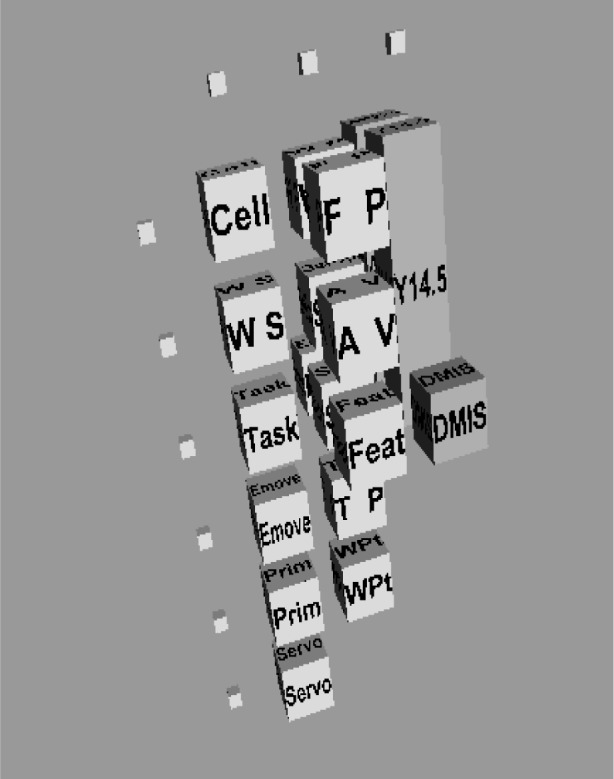
Inspection process as a 3D concept.

The representation for the inspection-process concept is formed by stacking the planes in Fig. 4 together into a 3D object as shown in Fig. 5. Note that the 2D boxes in Fig. 4 are represented by 3D boxes in Fig. 5 (with labels on every face), so that they can be seen as the planes are rotated. In addition, the small, unlabeled, white cubes in the figure are used to toggle the visibility of the concept planes on and off, as well as horizontal sections through them to assist in viewing selected parts of the object. Hence the 3D object gives the user intuitive and easy access to the dense information that is represented in the inspection process.

### 2.4 Interfaces

The inspection-process representation does not have sufficient resolution to compare information exchanges among different standards. Therefore it is used in concert with the interfaces concept. This concept, shown in Fig. 1, shows a perspective of the information exchange which is based on the functions involved rather than the control levels. These functions are considered from the perspective of producers or users of the information exchanged. For example, the “CAD” function produces data for functions at multiple levels of the control hierarchy. Conversely, the “Reporting and Analysis” function uses information retrieved from multiple sources up and down the control hierarchy.

This representation may be used in concert with the inspection-process representation to explore the information exchanges and compare the differences among the standards in a more intuitive manner than a mere text-based comparison. For example, one of the standards could be selected by clicking on it in the inspection-process representation and highlighting the information exchanges involved in the interfaces representation. Conversely, an information producer could be selected in the interfaces representation and the standards affected could be highlighted in the control hierarchy. Using this type of interplay between the two representations, and displaying information associated with the sub-objects upon command, the user can sift through the large base of data involved more intuitively and derive an enhanced understanding produced by seeing it in two different perspectives, that of the functions and interfaces and that of the control levels.

### 2.5 Inspection Device

A coordinate measuring machine (CMM) is an automated-inspection device. Seeing a simulation of an inspection rather than an abstract traversal of commands, statuses, and data flows through control levels can enhance the understanding of the whole process. It provides a different perspective of the same information which can appeal to the learning style of some students. Presenting the same information in different styles to a student can enhance the learning process.

The inspection-device concept is represented by a simple block model of a CMM that can be animated based on user interaction with additional controls and displays provided in the representation. The sub-components of the representation, beyond what is described here, can be used for knowledge associations that include different types of CMMs and probes, calibration knowledge, CMM error sources, etc. The CMM representation used in this project is a modified and enhanced version of a model that was originally created in an earlier project at NIST [8]. It should be noted that commercial systems exist that incorporate sophisticated models of CMMs.

### 2.6 Machining Errors

This concept covers errors that affect the part measurement results. Sources of machining errors include out-of-calibration machine tools, tool wear, and the environment (e.g., extreme temperatures). Ultimately, machining errors show up as deviations from the design dimensions of a part, based on the measured differences between the completed part and the nominal specification. The part would be incorrect if the differences caused an out-of-tolerance condition. The deviations will often show a distribution pattern that is characteristic of the type of process, the error source, and the type of surface being machined, e.g., a vertical end mill that was out of calibration could create lobing errors when machining out a hole. A taxonomy could be created that could connect particular manufacturing error sources to the error patterns created. Conversely, particular error results could be connected back to suspected error sources in the manufacturing process. Thus, a representation, as simple as a taxonomy, could be associated with an empirical database of the information described here.

## 3. Scenario

The idea envisioned is that the user would explore objects (concept representations), study the associated information, interact with the objects to gain intuitive insights, and observe the relationships among different objects. The exploration strategy outlined enables the user to construct an intuitive framework for a particular knowledge base in the user’s mind to support better understanding and retention of it.

This section provides the author’s conception for how this kind of system can be used to explore and study the knowledge base described in this paper. The treatment is brief and provides a flavor of the capabilities rather than a detailed description of them. In the scenario, two types of windows are referenced. The first is the concept window, referred to simply as a window, for display of concept representations. The second is the information window for display of information files or file fragments that are linked to the concepts’ sub-objects and accessed upon selection of them.

**Fig. 6:**
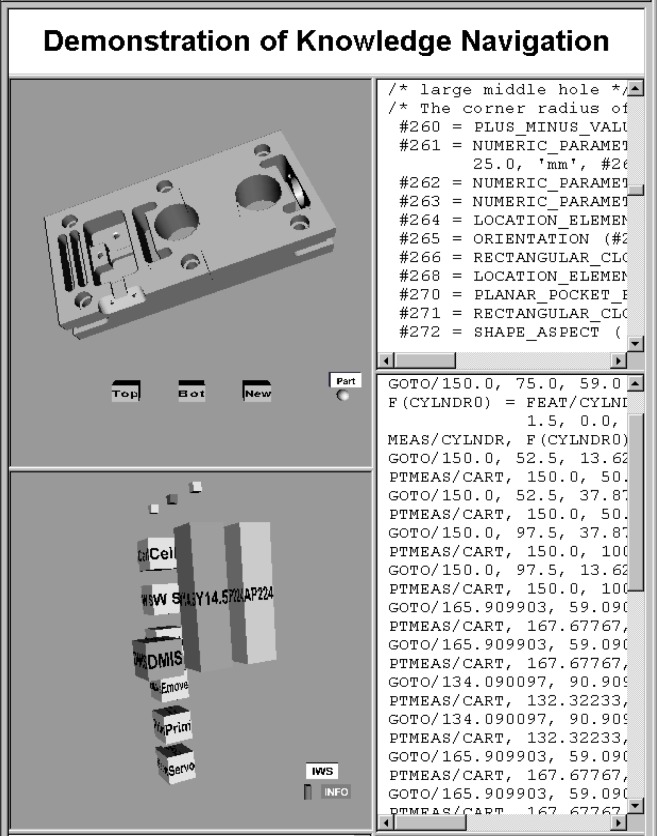
Screen view detail showing the part and inspection-process concepts, used to display part-feature information from text files. In figure, the text files are partially scrolled out of view.

For example, Fig. 6 shows a window with four frames. As mentioned earlier, a frame is referred to as a window in this paper. The two “windows” on the left are concept windows. The **part** concept is shown in the top-left window and the **inspection-process** concept is shown in the bottom-left window. The two windows on the right are information windows and contain file data that were sent to the respective windows as a result of the user clicking on features in the part concept. Also note that the middle concept plane of information types in the inspection-process concept has been rendered invisible to view the interoperability standards against the backdrop of the task decomposition.

The scenario below will first describe the exploration of the GD&T knowledge base; then it will describe the exploration of interoperability standards for the inspection process. Concepts selected by the user are shown in **boldface** in the discussion. Each concept can be set to MOVE mode in which case the user can manipulate the position and orientation of the concept representation, or it can be set to INFO mode in which case the user can access information by selecting a sub-object.

### 3.1 GD&T

The user calls up the **part** concept into a new window and manipulates it to see its features. Setting the mode to INFO, the mouse is moved over the part. When the mouse is over a feature, the feature is highlighted and a popup text lists a manufacturing process that could make the feature. Scanning the part in this way quickly reveals the types of processes that could make the part. If the mouse is clicked over a feature, a page of information is retrieved and sent to the information window that is currently active. That page first lists the manufacturing processes that could make that feature. (Usually more than one process could be used.) Each process identified is a link to further down the page where that process is described, and a discussion is included of why that process would be chosen for the feature selected.

The **machining-errors** concept is called up into a new window, and it shows a taxonomy of the type of error distributions that derive from different types of processes with different types of process anomalies. Based on the process chosen in **part**, taxonomy paths are highlighted to show the error sources possible for that process. Further information about the errors can be accessed by exploring the taxonomy and clicking sub-objects of it to call up information pages associated with those taxonomy items.

Going back to **part**, the user selects the GD&T option which causes the Y14.5 dimensions and tolerancing symbology to be visible on the part display. The symbology is connected to the 2D views of the part (top, front, etc.) and rotates along with the part as the part is rotated. If the front face is rotated to the back, the GD&T symbology associated with the front face is no longer visible. The user clicks on one of the tolerance feature control frames, and the **tolerance-entities** concept for that tolerance is called up into a new window.

The tolerance entities include the feature set toleranced (usually one feature), the datums, the tolerance zone and the feature control frame. The feature set shows the nominal surfaces and can be manipulated to see its various positions and orientations that will still keep it within the tolerance zone and consequently in tolerance. The tolerance value is connected to the tolerance zone and a change in either affects the other—e.g., for the flatness tolerance, increasing the tolerance value will cause the planes in the tolerance zone to move apart; conversely, moving the tolerance-zone planes closer together will cause the tolerance value to decrease.

Insights may be particularly valuable when looking at, for example, a positional tolerance for a feature that is located by one or more datums, and, additionally, the material-condition modifier is specified for either the feature or the datums (or both). For example, if a hole datum is modified by the maximum material condition (MMC), it affects the position of the tolerance zone of a hole that is referenced to that datum. By changing material conditions and tolerance values for different types of tolerances, considerable insights can be gained for the meaning of those tolerances.

Returning to the **machining-errors** concept, the error factors associated with different processes can be changed and the resultant error patterns displayed (as “whiskers” on a surface with the magnified lengths of each representing the deviation from the nominal surface). These error patterns can be sent to the **toleranceentities** concept to be superimposed on the feature toleranced. The user can obtain further insights by interacting with the tolerance entities as before and observing how different types of errors have different impacts on different tolerances. Note that the tolerance zone can be changed from invisible to opaque, the latter used to better observe the errors crossing the tolerance zone for out of tolerance conditions when the feature is “wiggled” to change its position and orientation.

### 3.2 Interoperability Standards

To explore interoperability standards the user calls up the **inspection-process** concept. By manipulating and scanning over the concept planes, the relationships of tasks, data, and standards can be observed in the context of the hierarchical control system for the inspection process. Clicking on any of the sub-concepts will call up a page of information about it; e.g., clicking on any of the standards will provide a page that briefly describes the standard. Clicking on an information block at the same level will show the types of information the standard will cover.

A full description of the information exchanges covered by the standard requires the user to call up the **interfaces** concept in a new window. When an interoperability standard is selected in the inspection process, the corresponding functions and information exchanges are highlighted in the interfaces concept. Clicking on any of the highlighted boxes or data arrows in that concept will show the information exchanged and its format for the standard selected. Calling up the **interfaces** concept again in a new window will put that concept in focus and a new standard selected in the **inspection process** will highlight the information exchanges for that standard in the new interfaces window. Then the standards can be compared side by side. Any information overlaps will be highlighted in a different color in both **interfaces** windows.

Finally, the user goes back to the **part** concept and sets its mode to INFO. When a part feature is selected, a data file or file fragment, associated with that feature and corresponding to the standard previously selected in the **inspection-process** concept, will be sent to the active information window. For example, if DMIS is selected in the inspection process, clicking on a part feature will send the DMIS file (of instructions to inspect that feature) to the active information window. By exploring interactively among these three concepts (inspection process, interfaces, and part) a large amount of information can be quickly explored and in a context to promote understanding and retention of it. Note that some standards affect other concepts. For example, DMIS commands can be executed by the CMM, an **inspection device**, and calling up that concept can further illustrate the information exchanges, what they mean, and how they are used.

## 4. Techniques

The approach and scenario can be implemented with the techniques described below.

### 4.1 Virtual Objects

The main interface to the knowledge base is a virtual object (in 2D or 3D) which is the representation of a concept. The object is decomposable into sub-objects, reflecting the concept’s decomposition, and the sub-objects are linked to information in the knowledge base. However, depending on the context of the concept, the sub-objects will be linked to different information. For example, assuming the concept is the part, the question could be asked, “what is the set of DMIS instructions to inspect one of the part’s features?” In this case, the part’s context is DMIS, and if a part’s feature is selected, a set of DMIS instructions to inspect that feature will be retrieved from the knowledge base and displayed. In other words, for each sub-concept, the corresponding sub-object will be linked to a number of difference pieces of information in the knowledge base, and the information retrieved when that sub-object is selected will be dependent on what context (or type) has been set for the parent object.

The virtual object might represent a physical object, e.g., a machined part; or it might represent an abstract concept, e.g., an inspection process where the separate applications of the process are represented by a set of boxes. For the latter case, the sub-objects are the boxes. However, a machined part is comprised of features that, generally, are created by volume-removal operations. In this case the features are the sub-objects. They may be the volumes removed, e.g., holes, or they may be the converse—what is left after material is removed, e.g., pegs.

The Virtual Reality Modeling Language (VRML) [9] was used to create the virtual objects in the demonstration prototype. VRML is not designed to represent a boolean subtraction (such as a volume removal). Therefore, for a physical object a technique was developed to superimpose a set of transparent surfaces that represent each feature on top of a solid model of the object. The information needed for a particular object is derived from a STEP AP224 file [10], which specifies the individual features of an object as well as the entire object.

When a sub-object is selected, two signals are sent out from the parent object. The first signal identifies the parent object and the second signal identifies the sub-object selected. A second object can be “wired” to receive those signals. That object can take two actions. It can set its information state, e.g., to specify what type of information to display when its own sub-objects are selected; or, it can take an action based on the signal, e.g., to display information based on the signal received and its previously set mode. Finally, all objects have a mode that is set to “MOVE” or “INFO.” In the MOVE mode, the object can be manipulated by the mouse (as described below under object manipulation). In the INFO mode, a mouse click on a sub-object will link to information that can be displayed in another window.

### 4.2 Multi-Modal Info Popup

When an object is in INFO mode, a mouseover of a sub-object will cause it to be highlighted and a popup caption, based on the information mode set, to be displayed. With this capability an object can be quickly scanned to see what types of information are associated with its sub-objects. For example, if the part object’s INFO type was set to “manufacturing process,” a mouseover of a feature would display a popup that listed a manufacturing process that could make it. If the feature was selected by a mouse click, detailed information about the manufacturing process associated with that feature could be displayed.

### 4.3 Knowledge Search

This system can be used in a manner analogous to accessing an information record from a database by specifying one or more of its key-field values. The concepts are analogous to the key fields, and each concept includes a range of values that can be selected (i.e., the sub-concepts). Several concepts can be called up and displayed. When the user selects a sub-object from one of the concepts, it is highlighted, and represents a key-field value that is shown in the context of the concept representation of which it is a component. Using this method, a search of the knowledge base can be specified and displayed on the screen as several concept representations with the appropriate sub-objects highlighted. These specify a particular information record in the knowledge base. Reiterating a point made previously, the degree of decomposition of concepts into sub-concepts determines the resolution of the system to distinguish among the items stored in the knowledge base.

### 4.4 User-Selectable Information Display

The goal is to allow the user to explore the knowledge base with the main attention focused on the task at hand to study the objects on the screen and pull up additional information as needed. The user should be able to direct that information to a window at any place on the screen, and have the flexibility to arrange multiple information windows as desired. At times the user might want to compare two pieces of information in two, side-by-side windows, e.g., two different representations of the same information from two different standards.

The flexibility described here should also apply to the virtual objects that represent concepts. The user should have the flexibility to place these in separate windows or group several of these objects in the same window. In either case, the objects should be able to communicate their signals to each other as specified above in “virtual objects.” The flexibility in placing these objects is partially enabled by the technique discussed next.

### 4.5 Object Manipulation and Visibility Controls

Object manipulation is necessary to position and orient a selected object (i.e., a concept) to access its sub-objects. Visibility control is used to selectively render certain groups of sub-objects invisible to reveal other sub-objects in a particular view that were previously hidden. The techniques employed should be easy to use and unobtrusive, so that the user can focus on the task at hand and not the controls.

To implement the first capability, a small “manipulation-control object” is placed near the object to be controlled. The control object is a small geometrical solid that is toggled to different shapes to represent different manipulations that the user invokes when dragging the main object with the mouse. For example, the control object can be programmed to toggle from sphere to cylinder to pointer and back again to sphere each time the user clicks on the control object. The three shapes correspond to a spherical rotation, cylindrical rotation, and a translation of the main object, respectively. Hence, if the manipulation control is set to a pointer, dragging on the main object will translate it to follow the mouse. In Fig. 6, the manipulation objects are located to the lower right of the part and inspection-process concepts. The manipulation objects can be moved within their respective windows by dragging their title bars.

The technique to selectively control the visibility of sub-objects was described in Sec. 2.3 when the implementation of concept planes for the inspection-process concept was discussed.

### 4.6 Concept Planes

This technique was described in Sec. 2.3.

## 5. Implementation

The computer system used for this work was a 300 MHz PC compatible with 128 MB of RAM. It would be considered at the low end for typical systems sold today. The system software included the Windows 95 Operating System and the Microsoft Internet Explorer 5.5 web browser.^2^

Because this project depends on 3D virtual objects, it was decided to use VRML to specify (i.e., model) them. VRML is a standard and a scene description language used to represent three-dimensional scenes that contain objects and their behaviors (including interactive behaviors among the objects as well as with the user) over the web. At the end of 1997, VRML97 became an official ISO standard [9]. To utilize VRML for this work a VRML plugin to the browser was installed. The plugin used was Cortona VRML Client 2.2 from Parallel-Graphics, available as a free download from their web site (www.parallelgraphics.com).

The software development system consisted of two tools, FrontPage 2000 from Microsoft Corporation for web page development and VrmlPad from Parallel-Graphics for creation of the VRML objects. The VRML objects included text, 3D objects, and 2D objects. JavaScript was used within the VRML Script Nodes to create the behaviors needed for the objects.

The demonstration prototype implements five of the six concepts introduced earlier, although not to the full extent described in Secs. 2 and 3. The concepts implemented are part, tolerance entities, inspection process, interfaces, and inspection device. The concept of machining errors was not implemented. Furthermore, the techniques described in Sec. 4 were not fully implemented. Notably, the ability to communicate from one concept (i.e., object) in a window to another concept in a different window (or different frame of the same window) was not implemented. Instead, to show how one concept can communicate to another, the demonstration prototype includes the example of the part concept and inspection-process concepts shown in the same window. When the user selects an interoperability standard in the inspection-process concept, it signals the part concept to set its information type to that standard, as described in the scenario in Sec. 3.2 for DMIS. In addition, the positional tolerance described in the scenario in Sec. 3.1, including the material conditions, was not implemented. Instead the simpler flatness tolerance was implemented. There are other differences that are not listed here.

## 6. Discussion

This section first discusses the novelty of this knowledge navigation system and then discusses the web technologies needed for implementing the techniques required.

### 6.1 Uniqueness of the Approach

This is a new approach to communicate a practical understanding of GD&T and the inspection process. Also, it includes a visualization of the coverage of information exchanges by interoperability standards for the inspection process. Some of these ideas have been used before. There are commercial products available that incorporate sophisticated 3D modeling, animation, and programming of the CMM inspection process. Other products provide online training for geometric dimensioning and tolerancing in a multi-media format. However, I do not know of any products that support the understanding of information exchanges as described by interoperability standards.

The novel aspects of this approach are listed below:

- organizing the dimensional metrology domain space into main concepts that are represented by virtual objects that can be manipulated and examined;
- the particular set of concepts chosen to represent a portion of the dimensional metrology domain;
- implementing the search of a knowledge base by the selection of key-field values that are displayed graphically as sub-concepts within the broader concepts to which they belong;
- the interactivity of the tolerance-entities concept and the combination of ideas it integrates together, i.e., how tolerance zones are affected by varying the tolerance value, the modifier (such as material condition) [11], and the type of error pattern (based on the manufacturing process);
- the approach of using AP224 to create transparent features that are superimposed on a 3D model that can be selected and linked to other information;
- the idea of concept planes to represent a multidimensional and dense set of information linkages in a 3D object;
- and finally, the implementation of the approach on the web using standardized technologies.

### 6.2 Web Technologies

The goal of this project is to use an open architecture, standards-based system for maximum dissemination. Consequently, this work has been made accessible via the web and uses techniques that employ standards that have been endorsed by the World Wide Web Consortium (W3C) [12].

As mentioned above, VRML is a standard that can be used to specify 3D objects. It can also be used to specify text and 2D objects. In fact, the capability in VRML to specify text, 2D, and 3D objects combined together in the same scene (and hence in the same window) was used in this project. However, it would also be useful to present some or all of the concept representations in their own windows. Then the screen space for the whole set of concepts displayed, including the additional information associated with various concepts and displayed in additional windows, could be managed with standard window functions. For example, the user could drag, scale, or stack the windows throughout the screen space as desired, and could rearrange those easily during a session. The problem is that, with the current technology, it is difficult, awkward, and unreliable to access an attribute of a VRML object in one window from another because multiple software interfaces need to be crossed to do so.

In addition, VRML runs in its own isolated environment. Models created elsewhere (e.g., HTML pages and 2D or 3D models) with other authoring applications cannot be simply accessed within a VRML application without further conversion or processing required. One solution, as implemented for this project, was to create the text and 2D objects with the VRML constructs available. The problem with this approach is that other applications specifically designed for creating text and 2D objects, e.g., the Y14.5 symbols, are easier to use and can produce more efficient structures for these types of objects than with the VRML constructs.

However, solutions are on the horizon, and emerging web technologies, including a next-generation VRML standard, should solve the problems mentioned above. The goal of the next generation of web standards and technologies will allow page elements (such as text, images, 2D and 3D objects, windows, frames, etc.) to be used very flexibly together, and all elements to be directly accessible through a script-based language such as JavaScript.

## 7. Conclusions and Future Work

This paper presents a new approach to convey understanding of dimensional metrology, in particular, geometric dimensioning and tolerancing and interoperability standards for the inspection process. The approach uses computer visualization to navigate a knowledge domain organized around key concepts represented by 2D and 3D virtual objects. Though the approach has been applied to dimensional metrology it can be applied to other domains as well; domains involving complex geometric entities and relationships are particularly well suited to this approach.

A demonstration prototype has been created to illustrate the ideas and techniques presented [13]. However, it has not been sufficiently developed to evaluate the approach for enhancing the understanding of dimensional metrology. Before that can be achieved, the techniques need to be reimplemented with emerging, standards-based, web technologies that will allow greater flexibility in integrating web-page elements that include text, 2D and 3D objects, and windows and window frames. In addition, sufficient content needs to be added to demonstrate a practical application. A full GD&T application would take considerable development work; there are many GD&T concepts to demonstrate and each one requires substantial design and development effort. Projecting further, a demonstration that shows the interactions of several tolerances of a part on each other would be very insightful to understanding GD&T but would be proportionately more difficult to implement. The application for interoperability standards needs further design and development to flesh out, but would be a considerably easier job than the GD&T application. Afterwards, comprehensive content would need to be added.

In closing, I recommend the development of a standardized taxonomy for dimensional metrology. That is an important step toward putting the subject online, because it would encourage the development of a distributed knowledge base that could be accessed by multiple applications, reducing the risk to instructional software developers. The ideas presented here for organizing the knowledge domain could be used to support that effort.

## Footnotes

1 For this paper, the term window refers to an internet-browser window or the frame of such a window.

2 Certain commercial equipment, instruments, or materials are identified in this paper to foster understanding. Such identification does not imply recommendation or endorsement by the National Institute of Standards and Technology, nor does it imply that the materials or equipment identified are necessarily the best available for the purpose.
